# Toward the Development of a Lupus Interactive Navigator to Facilitate Patients and Their Health Care Providers in the Management of Lupus: Results of Web-Based Surveys

**DOI:** 10.2196/resprot.3349

**Published:** 2014-12-22

**Authors:** Carolyn Neville, Deborah DaCosta, Murray Rochon, Davy Eng, Paul R Fortin

**Affiliations:** ^1^Division of Clinical EpidemiologyDepartment of MedicineMcGill University Health CentreMontreal, QCCanada; ^2^Jack Digital Productions Inc.Toronto, ONCanada; ^3^Centre de recherche du CHU de QuébecAxe maladies infectieuses et immunitairesQuebec, QCCanada; ^4^Division of RheumatologyDepartment of MedicineUniversité LavalQuebec, QCCanada

**Keywords:** systemic lupus erythematosus, needs assessment, access to information, self-management, patient navigator

## Abstract

**Background:**

Systemic lupus erythematosus is an inflammatory autoimmune disease associated with high morbidity and unacceptable mortality. Information and management tools are needed to help persons with lupus cope with their illness and facilitate health care providers in the delivery of care.

**Objective:**

The objective of the study was to assess the needs and find solutions to support persons with lupus and their health care providers.

**Methods:**

Web-based surveys were distributed across Canada to persons with lupus and their relatives (n=3119), rheumatologists (n=517), and arthritis health professionals (AHPs) (n=226) by Lupus Canada, the Canadian Rheumatology Association, and the Arthritis Health Professions Association, respectively.

**Results:**

The survey sample comprised 665 (21.3%) persons with lupus, 98 (19.0%) rheumatologists, and 74 (32.7%) AHPs. Among the participants with lupus, 92.4% were female, the average age was 46.8 (SD 12.7) years, 79.2% were Caucasian, and 58.8% were employed. All Canadian provinces and territories were represented. The majority (43.3%) of respondents were from Ontario. Mean disease duration was 10.2 (SD 9.5) years, and 41.9% rated their global assessment as fair or poor. There was high agreement between lupus participants and health care providers regarding disease-specific information topics. All groups rated topics related to lupus, fatigue, medications, and stress as most important. Ratings differed among lupus participants and their health care providers regarding perceived helpfulness of some of the patient tools, such as the option to view test results. Needs differed for persons with lupus based on age, sex, depression, stress, and disease activity. Differences in health care provider needs were based on amount of experience in treating lupus.

**Conclusions:**

Information and support tools needed for persons with lupus and their health care providers were identified. These results will help guide us in the development of a Web-based Lupus Interactive Navigator as an intervention tool to help persons with lupus self-manage their disease and to facilitate heath care providers in clinical management.

## Introduction

Systemic lupus erythematosus is a chronic autoimmune disease characterized by numerous clinical manifestations. The complexity of the illness and treatment creates serious burdens to both patients and health care providers. Challenges for persons with lupus are accepting the chronic and unpredictable nature of the illness, coping with their complex care, and accessing reliable information and resources with which to manage their illness. Adapted educational and support resources for persons with lupus are limited, difficult to access, and too often irrelevant to the reality of daily living with lupus. Information and support tools are needed to help persons with lupus self-manage their disease and help facilitate the delivery of optimal integrated care by health care providers.

**Figure 1:**
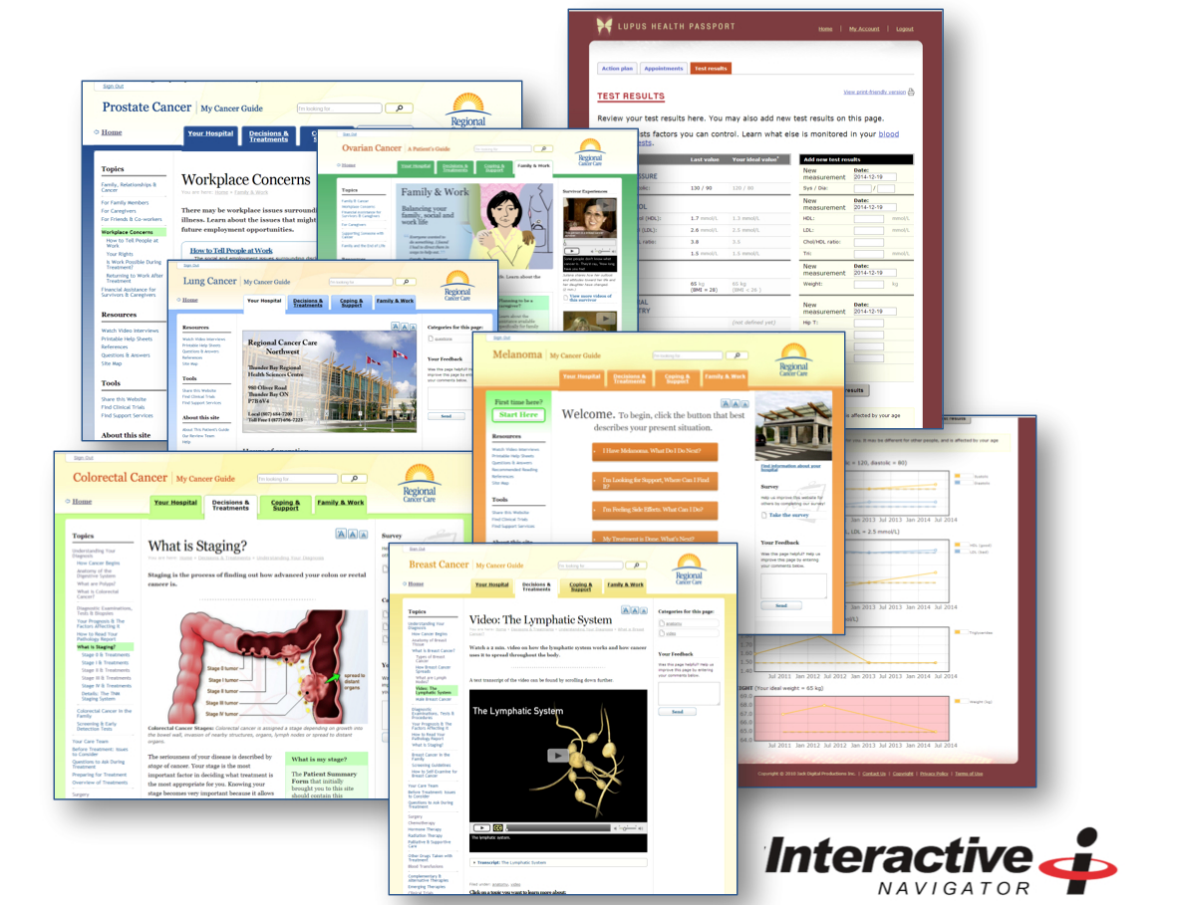
The Oncology Interactive Navigator is the prototype used for the development of the new Lupus Interactive Navigator. This figure illustrates some of the web-based pages of the Oncology Interactive Navigator.

Recently, a successful Web-based platform, the Oncology Interactive Navigator (Figure 1), was developed to provide information and tools to help persons with cancer manage their illness and become active participants in their health care [1,2]. On the basis of these findings related to that instrument, we are initiating the development of a virtual navigation tool adapted for persons with lupus, the Lupus Interactive Navigator (LIN), to provide these individuals with up-to-date information, tailor their access to appropriate health-related resources, and help them manage and cope with their disease. To this end, we conducted Web-based surveys and distributed them to persons with lupus, rheumatologists, and arthritis health professionals (AHPs) to assess their informational needs and to guide us in the development of the LIN. In this article, we report the results of the surveys.

## Methods

### Overview

Web-based surveys were distributed via FluidSurveys [3] to persons with lupus and their relatives (n=3119), rheumatologists (n=517), and AHPs (n=226) by Lupus Canada, which is a Canadian-based lupus patient/volunteer organization; the Canadian Rheumatology Association; and the Arthritis Health Professions Association of Canada, respectively.

### Survey Development

#### Content

The content of the surveys was established following discussions with members of an expert panel that included a rheumatologist, a psychologist, a nurse, a person with lupus, and the developer of the Oncology Interactive Navigator. This panel had previously been responsible for the development of focus group questions used in a complementary qualitative study on this topic [4]. Through a literature review and analysis of the results of the previous qualitative analysis, we identified informational needs of persons with lupus, rheumatologists, and AHPs, which led to our development of individualized surveys tailored for each group.

#### Persons With Lupus

For the persons with lupus, the surveys included questions designed to assess demographic and clinical characteristics, management strategies used, preferences regarding information topics, and tools to help manage lupus. Disease activity was assessed using a 10-point visual analog scale (VAS) (0=no activity; 10=most activity). Global assessment ratings were based on a 5-point Likert scale (1=excellent; 2=very good; 3=good; 4=fair; 5=poor).

Depression was assessed using the Patient Health Questionnaire-2 (PHQ-2) [5]. The PHQ-2 includes questions about the frequency of depressed mood and anhedonia over the previous 2 weeks and consists of 2 questions from the Patient Health Questionnaire-9 [6]. Each question is rated on a scale from 0 (not at all) to 3 (nearly every day). The PHQ-2 overall score ranges from 0 to 6. The intention of the PHQ-2 is not to establish a diagnosis, but to screen for depression. A PHQ-2 score ≥3 has a sensitivity of 83% and a specificity of 92% for detecting major depression [5]. Stress was assessed using the Perceived Stress Scale-4 (PSS-4) [7]. The PSS-4 assesses global perceived stress using 4 self-report items scored on a 4-point scale for a total possible score of 16 [8]. The response options for each item are as follows: 0=never; 1=almost never; 2=sometimes; 3=fairly often; 4=very often. The PSS-4 is not a diagnostic tool; it is used to compare stress levels within or between samples [7]. There are no established cutoffs for PSS-4. In accordance with previous work [9], we defined scores in the highest 2 quintiles as representing moderate to high stress.

Informational needs were assessed by asking participants to rate each item in a list of potential topics based on importance in managing lupus (1=least important; 10=most important). Management tool needs were assessed by asking participants to rate potential tools on how helpful they would be in disease management (1=least helpful; 10=most helpful).

#### Rheumatologists and Arthritis Health Professionals

Surveys for rheumatologists and AHPs included demographics (age, years in practice, and specialty), perceived barriers to providing health care for patients with lupus, and their preferences for patient information topics and tools. They were asked to rate patient information topics in terms of importance to their practice (1=least important; 10=most important) and patient management tools in terms of helpfulness to their practice (1=least helpful; 10=most helpful) in disease management. They were also asked to rate a series of clinical tools in terms of helpfulness to their practice (1=least helpful; 10=most helpful) in disease management.

#### All Groups

For all groups, ratings of ≥7 on individual items in the lists of information topics and tools were considered to be significantly important and helpful, respectively. Percentages were calculated based on the number of persons who rated individual items ≥7 in lists of information and tools.

### Statistical Analysis

The data were transferred from Fluid Surveys [3] to Microsoft Excel v.2007 files. Means, medians, and percentages were calculated for continuous values, and percentages were calculated for categorical values.

### Ethical Approval

Ethical approval to conduct the field surveys was obtained from the CHU de Québec Research Ethics Board.

## Results

### Persons With Lupus

#### Overview

The survey was mass-emailed to 3119 persons with lupus and their relatives, and we obtained a total of 808 respondents (25.90%). Of these respondents, 135 had no data related to lupus and were presumed to be relatives, and 8 were <18 years of age. We report the results of the 665 persons with lupus who responded (21.3% of original potential sample). Table 1 presents the characteristics of the persons with lupus.

All provinces and territories of Canada were represented, with the majority (43.3%) of respondents residing in Ontario. The majority (71.6%) lived in small to large urban communities, and 28.3% lived in small towns or rural communities. Among all the persons with lupus, 7.5% reported that the distance to the nearest regional hospital center was >80 km. Almost all persons with lupus (99.1%) had Web access, and most (85.5%) accessed the Web using personal computers. All respondents reported using the Web to access information about lupus, and 44.8% reported spending up to 5 h/wk for that purpose.

**Table 1:** Characteristics of the persons with lupus.

Characteristics			n (%)^a^ or mean (SD)
Consumers and relatives contacted			3119
Consumer responders (≥18 years)			665 (21.3)
Age (n=594), mean (SD)			46.8 (12.7)
Sex, n (percent female) (n=662)			612 (92.4)
Ethnic origin, n (percent Caucasian) (n=665)			527 (79.2)
**Marital status (n=664)**			
	Married and/or cohabiting		407 (61.3)
	Single		157 (23.6)
	Divorced		83 (12.5)
	Widowed		17 (2.5)
**Education (n=615)**			
	High school or less		138 (22.4)
	College/university		400 (65.0)
	Post-graduate/professional degree		77 (12.5)
**Employment (n=660)**			
	**Employed**		388 (58.8)
		Full-time	288 (43.6)
		
		Part-time	100 (15.2)
		Part-time due to SLE	60 (9.1)
	**Temporarily not employed**		25 (3.8)
	**Temporarily not employed due to SLE** ^b^		16 (2.4)
	**Not employed**		247 (37.4)
		Not employed due to SLE^b^	175 (26.5)
**Percent with access to the Web (n=650)**			644 (99.1)
	Work		306 (47.1)
	Home		598 (92.0)
	Library		58 (8.9)
	Other		44 (6.8)
**Device used to access the Web (n=641)**			
	Personal computer		548 (85.5)
	iPhone or other smartphone		93 (14.5)
**Web usage for information about SLE** ^b^ **(n=641)**			
	<1 h/wk		397 (61.9)
	1-5 h/wk		179 (27.9)
	6-10 h/wk		33 (5.1)
	>10 h/wk		32 (5.0)

^a^To adjust for missing values, percentages were calculated using non-missing values

^b^SLE: Systemic Lupus Erythematosus

#### Clinical Characteristics

The clinical characteristics of the persons with lupus are presented in Table 2. The average disease duration was 10.2 (9.5) years. The average disease activity score reported was 4.4 (2.8). Only 3% rated their global assessment as excellent; 55% rated it as good or very good; and 42% rated it as fair or poor.

The mean depression score on the PHQ-2 was 1.9 (1.8). The proportion of persons with lupus who screened positive for depression (score ≥3) was 28.1%. PSS-4 scores revealed that almost 50% of the persons with lupus scored in the top 2 quintiles. Of these, 17.4% scored in the 4th quintile, indicating moderate levels of stress, and 30.0% scored in the 5th quintile, indicating high levels of stress.

**Table 2:** Clinical characteristics of participants with lupus.

			n (%)^a^ or mean (SD)
Disease duration (y), mean (SD)			10.2 (9.5)
Disease activity^b^ , mean (SD)			4.4 (2.8)
**Global assessment (n=634), n (%)**			
		Poor	58 (9.1)
		Fair	208 (32.8)
		Good	236 (37.2)
		Very good	113 (17.8)
		Excellent	19 (3.0)
**PHQ-2 score (n=612), mean (SD)**			1.9 (1.8)
	Screened positive for depression^c^ , n (%)		172 (28.1)
**PSS-4 score (n=610), mean (SD)**			6.9 (3.1)
	Screened positive for moderate to severe stress^d^ , n (%)		291 (47.7)

^a^To adjust for missing values, percentages were calculated using non-missing values

^b^Disease activity was scored on a 10-point visual analog scale (0=no activity; 10=most activity)

^c^PHQ-2 score ≥3 indicates depression

^d^Scores in 4th and 5th quintiles on PSS-4 indicate moderate and high stress, respectively; PHQ-2: Patient Health Questionnaire-2; PSS-4: Perceived Stress Scale-4 (PSS-4)

#### Access to Health Care

The proportion of persons with lupus who reported having a family doctor was 93.1%. The distance from home to the family doctor varied from 1 to 2400 km, with a median of 30 km (IQR 15-77.5). Travel times from home to health care providers ranged from 0.2 to 15 hours, with a median of 0.75 hours (IQR 0.5-1.3). The proportion who reported having a rheumatologist or other lupus specialist was 87.5%. Distances traveled to a rheumatologist or lupus specialist ranged from 1 to 4000 km, with a median of 70 km (IQR 36.0-150). Travel times from home to a rheumatologist or lupus specialist ranged from 0.25 to 50 hours, with a median of 1.4 hours (IQR 1-2.5).

#### Self-Management Strategies Used

Exercise was the most frequently used management strategy (63.7%). Yoga (21.7%) and swimming (21.1%) were reported as helpful management strategies. Other frequently used management strategies reported to be helpful included prayer (42.5%), massage therapy (33.3%), and meditation (24.1%). Several strategies reported as less available but considered to be helpful were attending self-help groups; practicing stress management; and using community services, herbal medicine, and reflexology.

### Rheumatologists and Arthritis Health Professionals

#### Overview

Ninety-eight rheumatologists (19.0%) and 74 AHPs (32.7%) responded to the surveys. The average (SD) number of years in clinical practice reported by rheumatologists was 15.2 (12.0) (range, 6 months to 39 years). AHPs reported being in clinical practice for an average of 23.9 (12.7) years (range: 6 months to 45 years). Rheumatologists saw an average of 4.7 (4.6) patients with lupus per week (range, 0.2 to 20). Most rheumatologists (96%) reported that it would be beneficial to their practice if their patients with lupus played an active role in their own health care.

#### Barriers to Health Care

Rheumatologists rated patients’ non-adherence to medications as the greatest barrier to health care, with 76% rating it as problematic. Patients’ access to medications was also considered to be problematic (51.1%). Most problematic for AHPs were access to resources (81.9%) and patients’ non-adherence to treatments (75.4%).

#### Information Topics

The ratings of information topics for persons with lupus, rheumatologists, and AHPs are shown in Table 3. The topics were rated on a scale of 1 to 10 in terms of importance (1=least important; 10=most important), with percentages given for ratings ≥7.

The 3 most important information topics selected were similar across the 3 groups, with the general areas of fatigue management, understanding and coping with lupus, and medications most frequently being reported as important (84.8%-93.2%). Information about stress was rated important by slightly more persons with lupus (90.9%) and AHPs (84.7%) than rheumatologists (78.9%).

Most persons with lupus rated as important information topics related to self-management, including choosing options for living with lupus, coping with lupus, managing sleep disturbances, avoiding kidney disease, engaging in diet and exercise regimens, and managing pain (range, 80.2%-86.4%). Fewer rheumatologists rated these survey items as important (53%-66%), with the lowest percentage being for diet and exercise. Across the 3 groups, the information topics least often rated as important addressed disability insurance (48.3%-55.0%) and employment counseling (41.8%-64.4%).

Surprisingly, access to psychosocial resources was rated as important less often by persons with lupus (52.6%) than by rheumatologists (79.7%) and AHPs (86.4%). Not surprisingly, information about complementary and alternative therapies was rated as important more often by persons with lupus (68.7%) than by rheumatologists (38.5%) and AHPs (32.8%).

**Table 3:** Percentage reporting individual information topics as important.^a^

		Persons with lupus	Rheumatologists	AHPs
Dealing with fatigue		91.3	84.8	93.2
Understanding lupus, the disease		90.9	86.1	91.4
Understanding effects of stress on lupus		90.9	78.9	84.7
Medications used in lupus		88.7	92.4	87.9
Practical lifestyle options for living with lupus		86.4	N/A^c^	N/A
Managing sleep disturbances		85.1	N/A	N/A
**Diet and exercise recommendations** ^b^		83.4		
	Diet recommendations		53.1	58.6
	Exercise recommendations		61.5	77.6
Coping with arthritis		81.3	N/A	N/A
Avoiding kidney disease		81.2	N/A	N/A
Pain management		80.2	65.8	89.8
Decision-making information		74.7	N/A	N/A
Addressing depression		73.2	69.2	89.8
Managing skin rashes		69.8	69.6	71.1
Improving communication with the health care team		68.8	64.6	66.1
Complementary and alternative methods		68.7	38.5	32.8
Knowing where to get disability insurance		55.0	50.0	48.3
Access to psychosocial resources		52.6	79.7	86.4
Employment counseling services		41.8	48.1	64.4

^a^Percentages are for ratings ≥7 in terms of importance (1=least important; 10=most important)

^b^Diet and exercise recommendations were combined in the survey for persons with lupus

^c^N/A=Not Asked

### Management Tools for Persons With Lupus

The ratings of the patient management tools are shown in Table 4. Management tools for persons with lupus were rated on a scale of 1 to 10 in terms of importance (1=least important; 10=most important), with percentages given for ratings ≥7.

More persons with lupus (87.5%) than rheumatologists (59.6%) rated the helpfulness of the option to view test results in managing lupus as important. Similar numbers of persons with lupus and rheumatologists rated options to update medical information (78.5% and 70.9%, respectively) and review and update medications (75.7% and 70.9%, respectively) as helpful. Coping tools, such as journaling to record symptoms and flares and to track mood and stress, were viewed as helpful more frequently by persons with lupus (70%-72%) and AHPs (71%-74%) than by rheumatologists (39%-53%). Similar numbers of persons with lupus (65.3%) and rheumatologists (62.0%) reported that a community resource locator would be helpful. Fewer than half of persons with lupus rated chat rooms and prednisone-tapering calendars as helpful.

**Table 4:** Percentage reporting individual management tools as helpful. ^a^

Management tools	Persons with lupus	Rheumatologists	AHPs
Option to view test results	87.5	59.6	N/A^b^
Option to update medical information	78.5	70.9	N/A
Monitor emotional wellness	76.8	N/A	N/A
Option to review and update medications	75.7	70.9	N/A
Journal symptoms and flares	72.1	53.2	70.7
Track mood/stress levels	69.9	39.2	74.1
Community resource locator	65.3	62.0	84.5
Chat rooms	53.8	N/A	N/A
Calendars specific to prednisone tapering	41.5	N/A	N/A

^a^Percentages are for ratings ≥7 in terms of importance (1=least important; 10=most important)

^b^N/A: Not Asked

### Clinical Tools

The results of rheumatologist and AHP ratings of the helpfulness of clinical tools are shown in Table 5. Among the clinical tools listed in the survey, rheumatologists most frequently rated as helpful patient reminders for screening and vaccinations (88.3%), current medication lists (85.5%), printer-friendly patient information (84.2%), and access to view medication changes made by the another physician or by the patient (84.2%). Other tools that they considered to be helpful were printer-friendly prednisone-tapering schedules (76.6%), access to tests results (73.1%), links to patients’ general practitioners and specialists (72.7%), and access to anthropomorphic and clinical measures (68.8%). The fewest rheumatologists reported 36-Item Short Form Health Survey (SF-36) [10] scores (34.2%), template referral letters (49.4%), and ability to correspond with patients (55.8%) as helpful.

All AHPs rated printer-friendly patient information as helpful. Other items many AHPs rated as helpful were links to resources (93.1%), links to general practitioners and specialists (87.7%), and ability to correspond with patients (84.2%). Slightly more than half (54.4%) of them rated SF-36 scores as important.

**Table 5:** Percentage of rheumatologists and arthritis health professionals rating of individual clinical tools as helpful.^a^

Clinical tools	Rheumatologists	AHPs
Reminders (screening, vaccinations, etc.)	88.3	N/A^b^
List of patients’ current medications	85.5	
Printer-friendly patient information (e.g., medication instructions)	84.2	100.0
Access to view medication changes made by another MD or the patient	84.2	N/A
Printer-friendly prednisone-tapering schedules	76.6	N/A
Access to tests results	73.1	N/A
Links to GPs^c^ and specialists	72.7	87.7
Anthropomorphic and clinical measures	68.8	N/A
Links to resources	63.6	93.1
Access to LIN^d^ to view and update medical data	61.8	N/A
SLEDAI^e^ and SLICC DI^f^ scores	61.0	N/A
Ability to correspond with patients to provide reminders and answer questions	55.8	84.2
Template referral letters	49.4	N/A
SF-36^g^ scores	34.2	54.4

^a^Percentages are for ratings ≥7 in terms of importance (1=least important; 10=most important)

^b^N/A: Not Asked

^c^GP: General Practitioner

^d^LIN: Lupus Interactive Navigator

^e^SLEDAI: Systemic Lupus Erythematosus Disease Activity Index [11]

^f^SLICC DI: Systemic Lupus Erythematosus Collaborating Clinics Disease Index [12]

^g^SF-36: 36-Item Short Form Health Survey

### Differences in Needs Based on Characteristics of Respondents

#### Persons With Lupus

We further evaluated whether the differences in ratings were due to respondents’ characteristics.

We evaluated the responses of persons with lupus by individual characteristics to determine whether ratings differed by age; sex; disease duration; and disease activity, depression, and stress scores.

We found similar ratings among the persons with lupus aged <40 years and those aged ≥40 years regarding all information topics. However, the persons with lupus aged ≥40 years placed importance on management tools such as chat rooms and prednisone calendars less frequently than the younger participants did. Men less frequently than women placed importance on complementary and alternative therapies (47.9% versus 69.7%, respectively), journaling symptoms (56.3% versus 73.8%, respectively), chat rooms (43.8% versus 54.8%, respectively), and resource locators (55.3% versus 66.2%, respectively). Disease duration had no impact on ratings for information topics and management tools.

We found numerous differences in ratings between those with low disease activity (VAS score <5) and those with high disease activity (VAS score≥5). Participants with high disease activity more frequently reported interest in self-management topics, including depression, coping, sleep, pain, disability, psychosocial resources, and improving communication with the health care team. Also, compared to persons with low disease activity, those with high disease activity more frequently rated as important management tools such as prednisone calendars and journaling. Patients with greater lupus disease activity scored higher for depression on the PHQ-2 depression scale than those with lower lupus disease activity (37.4% versus 18.4%).

On all items, persons with lupus who screened positive for depressed mood (PHQ-2 score ≥3) were more likely than those without depressed mood to rate items as important. The greatest differences occurred with regard to topics related to depression, sleep, pain, disability, decision making, and psychosocial and community resources. Persons with lupus and depressed mood scored these items 8%-22% higher than persons with lupus who did not have depression. The greatest differences in ratings of the helpfulness of management tools were for chat rooms, tools to track mood and stress levels, and tools to monitor emotional wellness. Persons with lupus and depressed mood rated these items 8%-16% higher than those who were not depressed.

Participants with lupus who screened positive for moderate or severe stress rated most items somewhat higher (by 3%-5%) compared to those who were less stressed.

#### Rheumatologists

Numerous differences were observed regarding responses of rheumatologists with relatively more experience in treating patients with lupus (>5 patients per week versus ≤5 patients per week). Rheumatologists with relatively more experience rated most items higher than those who were treating fewer patients. The greatest differences were in patient self-management topics, including exercise, stress management, depression, and rash management, with more experienced rheumatologists rating these items 6%-14% higher than those treating fewer patients. Also, the more experienced rheumatologists placed more importance on patient management tools, including patient access to records to update medical information and medications. Differences between more and less experienced rheumatologists were also observed with regard to clinical management tools. Compared to rheumatologists who treated fewer patients with lupus, those with more experience gave higher ratings to clinical management tools such as access to test results, SLEDAI and SLICC DI scores, and ability to correspond with patients.

## Discussion

### Principal Findings

To our knowledge, this is the first large, comprehensive survey of persons with lupus and their health care providers conducted to date to identify information and tools needed to help these patients with self-management of their disease and to facilitate clinical management. The demographic and clinical characteristics of the persons with lupus were similar to the average Canadian lupus patient population and provided a wide spectrum of disease duration (0.7-19.7 years) and disease activity and global assessment ratings.

The following limitations of our study design should be noted. The surveys were distributed to persons affiliated with Lupus Canada who had Web access. The findings may not reflect the needs of persons with lupus who are not members of Lupus Canada or do not use computers. Also, although the survey was offered in both English and French, there were very few French-speaking respondents and thus, the needs of French-speaking persons with lupus were underreported.

The fact that we received responses from more than 600 persons with lupus indicates that a large number of consumers use email as a means of communication. This finding supports the feasibility of using a Web-based program to reach large numbers of persons with lupus. However, we do not know the prevalence of persons with lupus who do not have Web access; therefore, we cannot generalize our findings to all persons with lupus in Canada. Among the entire population in Canada, 83% reported having access to the Web [13].

Depressed mood and stress were present in some of the persons with lupus in our sample, with 28.0% having PSQ-2 scores suggesting depressive disorder and 49.9% with PPS-4 scores suggesting moderate to severe stress. Although the PHQ-2 is a screening tool, not a diagnostic tool, it has previously been shown to be a valid and reliable instrument [5,14] that can easily be used for research and in clinical practice owing to its brevity. The prevalence of depression in lupus is high and has been shown to be as high as 39% [15]. Our findings suggest that these brief measures may be useful in identifying those persons with lupus at high risk for depression or elevated stress and point to a need for use of an instrument such as the LIN to address mental health issues and guide the selection of resources to help manage depression and stress.

The most important information topics across all groups dealt with medications, fatigue, and management of lupus. Persons with lupus placed somewhat higher importance on disease management information, including stress and pain management, diet, and exercise, than rheumatologists did. However, the more experienced rheumatologists gave higher ratings for stress and exercise than rheumatologists who followed fewer patients. These results indicate that rheumatologists who treat relatively more patients with lupus, compared to those with less experience, are more in tune with these patients’ needs.

The greatest differences in ratings observed between rheumatologists and persons with lupus were for information topics about complementary and alternative therapies and access to psychosocial resources. It is not surprising that persons with lupus placed higher importance on complementary and alternative therapies than rheumatologists did. Patients with chronic diseases have been shown to seek these therapies frequently [16]. The opinions of rheumatologists regarding use of complementary and alternative therapies may be driven by the lack of proven scientific evidence for most of them [17]. We were surprised to see that rheumatologists’ ratings for information about access to psychosocial resources were higher than those of persons with lupus. These results may reflect rheumatologists’ increasing awareness of the psychosocial burden on their patients and in their clinical practice. Rheumatologists have reported the lack of these resources to be one of the barriers to health care. Rheumatologists are not prepared to provide psychological help and do not have the time to address these needs. Patient access to psychosocial resources would benefit their patients and greatly relieve their clinical workload.

There were also several differences between rheumatologists and patients with regard to ratings of the helpfulness of patient tools, including options to view test results, journaling symptoms and flares, and tracking mood and stress levels, with rheumatologists rating these tools as less helpful than patients did. There remained a large difference in these ratings regardless of rheumatologists’ experience levels. The ratings for the option to view test results may reflect rheumatologists’ concerns that patients with lupus could become anxious and overwhelmed if not prepared with adequate information about test results and medical data. It is unclear why rheumatologists did not perceive patient options to track mood and stress and journal symptoms and flares as helpful to their practice. Perhaps the tracking and journaling options were considered to be less clinical and to be more helpful in psychological therapy than in medical practice.

The clinical tools that rheumatologists considered most beneficial to their practice were options to remind patients about screening and vaccinations, current medication lists of their patients, printer-friendly patient information, and access to view medication changes made by patients or by other physicians.

### Conclusions

In this study, we identified specific informational needs and tools to help persons with lupus and their health care providers better manage lupus. Furthermore, we identified needs specific to persons with lupus based on their characteristics.

There was high agreement between persons with lupus and rheumatologists regarding disease-specific information topics. Although rheumatologists placed somewhat lower ratings of importance on topics related to information on patient lifestyle choices and self-management tools, their ratings did reflect that they felt these areas have some importance. Furthermore, rheumatologists who were more experienced in treating patients with lupus placed higher importance on some of the self-management information topics (eg, exercise, managing stress, depression) and self-management tools, including patient access to update medical information and medications. In future studies, we will focus on the topics of greatest importance to persons with lupus and their health care providers and will further tailor the LIN to the specific needs of persons with lupus based on these individuals’ characteristics, including depression and level of disease activity, to best serve their needs.

## Abbreviations

AHPs: Arthritis health professionals
CIHR: Canadian Institute of Health Research
LIN: Lupus Interactive Navigator
JDP: Jack Digital Productions Inc
PHSI: Partnership in Health System and Improvement
PHQ-2: Patient Health Questionnaire-2
PSS-4: Perceived Stress Scale-4
SLEDAI: Systemic lupus erythematosus disease activity index
SLICC DI: Systemic Lupus International Collaborating Clinics/American College of Rheumatology Damage Index
SF-36: 36-Item Short Form Health Survey
VAS: Visual analog scale
